# Data-independent acquisition mass spectrometry identification of extracellular vesicle biomarkers for gastric adenocarcinoma

**DOI:** 10.3389/fonc.2022.1051450

**Published:** 2022-11-24

**Authors:** Lei Gu, Jin Chen, Yueying Yang, Yunpeng Zhang, Yuying Tian, Jinhua Jiang, Donglei Zhou, Lujian Liao

**Affiliations:** ^1^ Department of General Surgery, Shanghai Tenth People’s Hospital, School of Medicine, Tongji University, Shanghai, China; ^2^ Shanghai Key Laboratory of Regulatory Biology, School of Life Sciences, East China Normal University, Shanghai, China; ^3^ Department of Interventional Oncology, Renji Hospital, Shanghai Jiaotong University School of Medicine, Shanghai, China; ^4^ Department of Gastric Surgery, Fudan University Shanghai Cancer Center, Shanghai, China; ^5^ Department of Oncology, Shanghai Medical College, Fudan University, Shanghai, China; ^6^ Durbrain Medical Laboratory, Hangzhou, Zhejiang, China

**Keywords:** proteomics, serum, extracellular vesicle, gastric adenocarcinoma, biomarker

## Abstract

Early diagnosis of gastric adenocarcinoma (GAC) can effectively prevent the progression of the disease and significantly improve patient survival. Currently, protein markers in clinical practice barely meet patient needs; it is therefore imperative to develop new diagnostic biomarkers with high sensitivity and specificity. In this study, we extracted extracellular vesicles (EV) from the sera of 33 patients with GAC and 19 healthy controls, then applied data-independent acquisition (DIA) mass spectrometry to measure protein expression profiles. Differential protein expression analysis identified 23 proteins showing expression patterns across different cancer stages, from which 15 proteins were selected as candidate biomarkers for GAC diagnosis. From this subset of 15 proteins, up to 6 proteins were iteratively selected as features and logistic regression was used to distinguish patients from healthy controls. Furthermore, serum-derived EV from a new cohort of 12 patients with gastric cancer and 18 healthy controls were quantified using the same method. A classification panel consisting of GSN, HP, ORM1, PIGR, and TFRC showed the best performance, with a sensitivity and negative predictive value (NPV) of 0.83 and 0.82. The area under curve (AUC) of the receiver operating characteristic (ROC) is 0.80. Finally, to facilitate the diagnosis of advanced stage GAC, we identified a 3-protein panel consisting of LYZ, SAA1, and F12 that showed reasonably good performance with an AUC of 0.83 in the validation dataset. In conclusion, we identified new protein biomarker panels from serum EVs for early diagnosis of gastric cancer that worth further validation.

## Introduction

Gastric adenocarcinoma (GAC) is a highly invasive cancer with the third highest mortality rate world-wide (1–3). Although technology advancements have reduced the overall incidences and mortality of GAC (2), it remains one of the most common cancers (4). This situation is partly due to lack of sensitive methods to identify GAC at early stages, leading to unnoticed tumor progression and poor patient prognosis. Thus, early diagnosis of GAC has the potential to greatly improve the chance of patient survival (5).

Diagnostic methods used in clinical practice include upper gastrointestinal (UGI) radiography, endoscopy, histopathology and liquid biopsy. Endoscopy-guided biopsy and pathology is the “gold standard”, and is necessary for confirming the malignancy, stage, and tissue of origin (6). However, due to its invasive nature and poor patient compliance, extensive use of endoscopy in screening GAC is impractical. On the other hand, the emerging technology of liquid biopsy features non-invasiveness and low cost and has the potential to provide diagnostic information prior to the onset of symptoms, could provide a promising tool for screening gastric cancer at early stages. As potentially valuable diagnostic tools, individual protein markers provide relatively low sensitivity and specificity at present. For example, the sensitivity of CA72-4, CEA and CA125 in detecting GAC are all below 40%, but the sensitivity of combining the three proteins can rise to 66% (7). Still, sensitivity at this level remains too low to satisfy clinical demands. Discovering more efficient protein panels holds the promise to improve the sensitivity of detecting cancer at an early stage.

Previous studies have shown that EV is involved in many processes in the onset and development of gastric cancer (8–10). These vesicles carry RNAs, proteins as well as metabolites, which may reflect the pathological state of cancer cells. EV can transport specific proteins and nucleic acids into target cells in the tumor microenvironment, affecting tumor cell proliferation and metastasis, inhibiting immune surveillance and incurring drug resistance (11). In addition, the membrane structure of EV can preserve the molecular components. Due to its versatile functionalities, EV has caught tremendous attentions in cancer field.

In this study, we extracted EV from sera of GAC patients and healthy subjects, and applied LC-MS/MS technology to capture protein expression profiles. From this dataset we further screened reliable diagnostic protein biomarkers for GAC, aiming to explore the clinical usefulness of serum EV.

## Materials and methods

### Collection of serum samples

Peripheral blood samples were collected from healthy controls and GAC patients at Shanghai Tenth People’s Hospital. Cohort 1 consisted of 19 controls and 33 patients, while Cohort 2 consisted of 18 controls and 12 GAC patients. The demographic data and staging information are listed in **Table 1**. To prepare serum samples, venal blood was drawn and placed at room temperature for 30 minutes, then centrifuged at 2000 × *g* for 10 min. Serum was collected and stored at -80°C until use.

**Table 1:** Clinical information (X denotes information unavailable).

Characteristic	Cohort 1		Cohort 2		Total
	Healthy	Gastricadenocarcinoma	Healthy	Gastricadenocarcinoma	
**Total cases**	19	33	18	12	82
**Age (years)**					
Median	55	65	63	68	62
Range	27-74	35-82	46-75	56-85	27-85
**Sex**					
Male	9	26	9	6	50
Female	10	7	9	6	32
**Stage**					
I	–	8	–	1	9
II	–	9	–	3	12
III	–	13	–	7	20
IV	–	3	–	1	4
**T**					
1	–	6	–	1	7
2	–	2	–	0	2
3	–	13	–	5	18
4	–	11	–	5	16
X	–	1	–	1	2
**N**					
0	–	14	–	4	18
1	–	3	–	2	5
2	–	5	–	4	9
3	–	8	–	1	9
X	–	3	–	1	4
**M**					
0	–	30	–	11	41
1	–	3	–	1	4

### Isolation of EV from serum samples

EV was isolated from serum samples using ultracentrifugation (UC). Briefly, 500 μL of serum was centrifuged at 2500 × *g* for 10 min (4°C) followed by another centrifugation at 10000 × *g* for 30 min to pellet cell debris. The supernatant was then filtered through a 0.22-μm cellulose acetate centrifuge filter (Costar, USA), and the filtrate was diluted with PBS into a final volume of 5 mL. Crude EVs were pelleted by ultracentrifugation at 110,000 × *g* (P70AT rotor, Hitachi, Japan) for 5 h. Afterwards, the pellets were resuspended with PBS and ultracentrifuge at 110,000 × *g* for 70 min. The final EV pellets were resuspended with 50 μL of PBS and stored at -80°C for further analysis.

### Characterization of EVs

For transmission election microscopy (TEM), 10 μL of PBS-diluted EV samples were added on top of the copper grids and incubated for 10 min at room temperature. The grids were washed with 6 μL of ultrapure water and negatively stained with 3% phosphotungstic acid for 10 min. Then, the grids were washed with ultrapure water and air-dried. Imaging was performed on a H-7700 transmission election microscope (Hitachi, Japan).

To analyze particle size, EV samples were diluted 10 times with PBS and analyzed with Zetasizer Nano S instrument (Malvern, UK) according to manufacturer’s instruction.

EV samples were lysed with RIPA buffer (150 mM NaCl, 0.5% SDC, 0.1% SDS, 50 mM Tris/HCl, 1% Triton-X 100, pH 7.6) and 20 μL of each lysed sample was separated on 12% SDS-PAGE. For immunoblotting, proteins were transferred onto a nitrocellulose membrane and incubated with anti-CD9 antibody (1:1000; Cat. #ab92726, Abcam, UK) and anti-Hsp70 antibody (1:1000; Cat. #ab181606, Abcam, UK) followed by anti-rabbit IgG secondary antibody (1:10000; Cat. #09-0034, Yeason, China). For silver staining, samples were washed with 50% methanol, 5% methanol and pure water successively, and then reduced by 0.0005% dithiothreitol (DTT; Cat. #43217, Sigma-Aldrich, USA) followed by incubation with 0.1% silver nitrate in the dark for 20 min. Finally, 3% sodium carbonate with 0.01% formaldehyde was applied for visualization.

### Protein digestion

BCA kit (Cat. #23225, Thermo Science, USA) was used to determine the protein concentration in isolated EVs samples. From each sample, 20 μg protein was vacuum dried and resuspended in denaturing solution (7 M urea, 2 M thiourea, 10 mM DTT, 1 × protease inhibitor [Cat. # P8340, Sigma-Aldrich, USA]). The samples were then reduced for 30 min at 55°C, alkylated with 15 mM iodoacetamide (Cat. # I1149, Sigma-Aldrich, USA) in the dark for 20 min, diluted with 50 mM ammonium bicarbonate solution and digested with trypsin (1:50; Cat. # V5113, Promega, USA) overnight at 37°C. The resulting peptides were desalted with C18 column and vacuum dried for mass spectrometry analysis.

### LC-MS/MS analysis

Protein digests were analyzed on an EASY-nLC 1000 LC (Thermo Science, USA) coupled with Q-Exactive mass spectrometer (Thermo Science, USA). The mobile phases consisted of buffer A (2% ACN, 0.1% formic acid) and buffer B (98% ACN, 0.1% formic acid). Tryptic peptides were resuspended in buffer A and spiked with iRT peptides (Omicsolution, China). Equivalent to 1 μg of protein digest from each sample was loaded onto a C18 column (Cat. #164534, Thermo Science, USA) linked with a pre-column (Cat. #164535, Thermo Science, USA) and separated at a flow rate of 250 nL/min. A 120 min gradient from 3% to 8% buffer B in 5 min, 8% to 28% in 95 min, 28% to 95% in 10 min, 95% for 5 min, 95% to 3% in 2 min and 3% for 3 min was used. The MS instrument was operated in the positive polarity and profile mode with a nano-electrospray through a heated ion transfer tube with a temperature setting of 275°C. For data dependent acquisition (DDA), one full scan MS from 400 to 1400 m/z followed by 12 MS2 scan were continuously acquired. MS spectra were acquired with resolution of 70000 for a maximum injection time (IT) of 100 ms with an automatic gain control (AGC) target value of 3e6. MS2 spectra were obtained in the higher-energy collisional dissociation (HCD) mode using a normalized collision energy of 27%, resolution at 17500, maximum IT of 60 ms, AGC target of 5e5 and isolation window at 2.0 m/z. For data independent acquisition (DIA), isolation window for MS2 was set to 20 Da with 1 Da overlap over a precursor mass window of 450~950 m/z, and other parameters were set to be the same as DDA method.

### Analysis of proteomic data

Qualitative analysis of DDA raw files was performed by Proteome Discoverer (version 2.0) software searching against the UniProtKB database (2020 release, *Homo sapiens*) including the 11 synthetic iRT peptides. A maximum of 2 missed cleavages were allowed for trypsin digestion with fixed carbamidomethylation (+57.0251 Da) of cysteine and oxidation (+15.9949 Da) of methionine. The mass tolerance allowed was 15 ppm for precursor ions and 0.05 Da for fragmentation ions. A false discovery rate (FDR) of 1% was set at both peptide and protein levels.

Skyline (version 20.2.0.343) software was used for independent proteome spectral library construction. Briefly, fasta and pdresult files were imported with the following parameters: structural modifications: carbamidomethyl (C), oxidation (M), acetyl (N-term); minimum length: 6; maximum length: 30. The retention time of iRT was calibrated and the isolation scheme was set based on the isolation window of DIA MS parameter. Target list was added to obtain information of peptides and proteins. Finally, the peptides with reversed sequence were added as decoy peptides for the control of false discovery rate.

DIA raw files were imported into skyline and analyzed based on the aforementioned DDA spectral library, and filtered by a mProphet scoring model trained with decoy peptides. Q value and dot products were set to 0.01 and 0.65 respectively for selecting peptides with high confidence. Afterwards, the decoy peptides were removed. The exported files were used for statistical analysis.

### Statistical and bioinformatic analysis

RStudio (version 1.3.1073) was used to perform all the statistical analysis, including evaluation of data quality, data preprocessing, differential expression analysis, principal component analysis (PCA), construction of classification models. For differential expression analysis, p value < 0.05 were considered statistically significant, and fold change > 1.5 or < 0.67 were considered as up- or down-regulated, respectively. Venn diagram and functional annotation were generated with FunRich (version 3.3.1) software.

## Results

### Study design and characterization of EV

**Figure 1:**
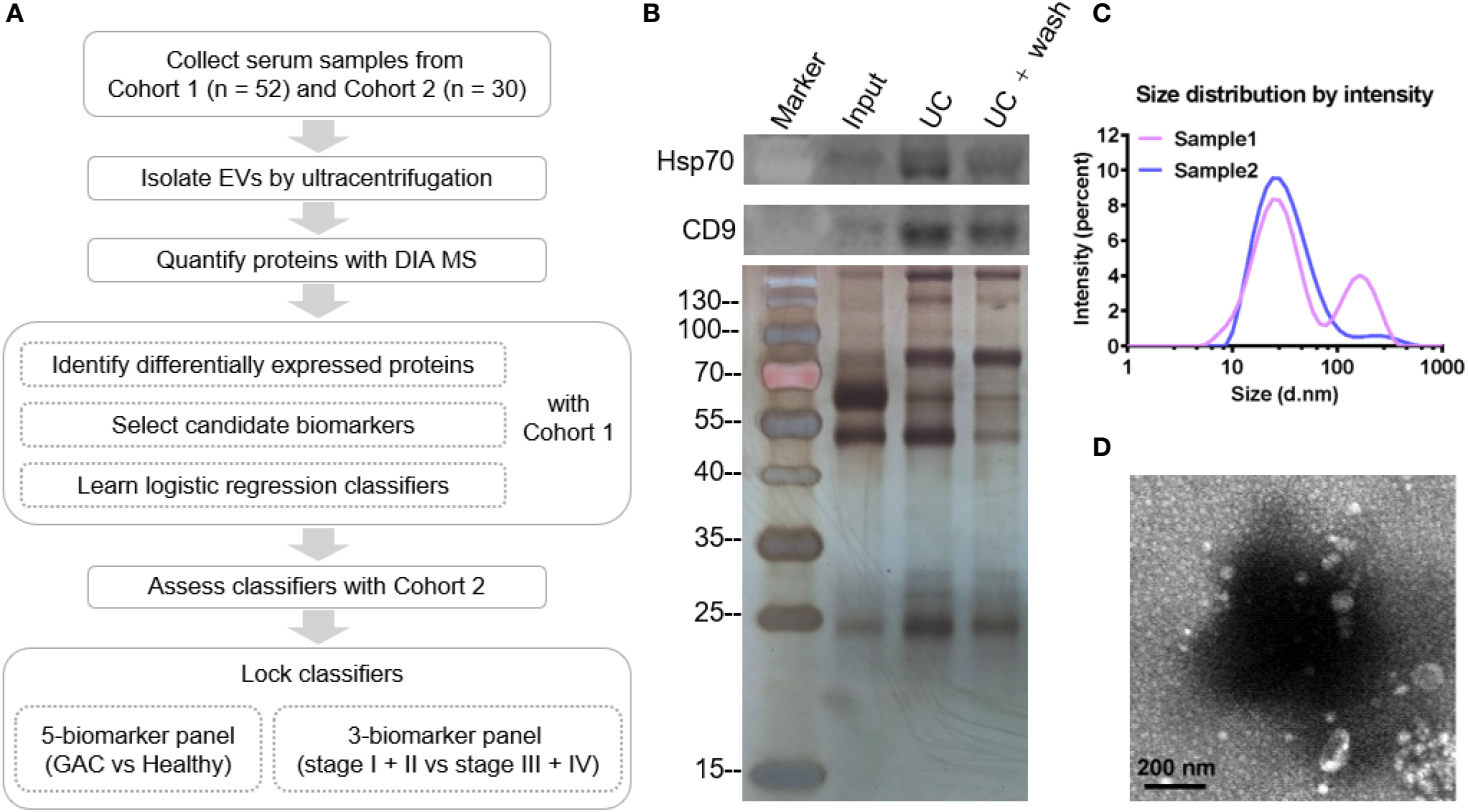
Design and quality assessment of isolated serum EV. **(A)** Flow chart displaying the study design of this study. **(B)** Western blot of EV samples showing Hsp70 and CD9, and SDS-PAGE of EV samples followed by silver stain. Input: serum; UC, ultracentrifugation. **(C)** Particle size distribution of isolated serum EV. **(D)** Representative TEM image of isolated EV. Scale bar: 200 nm.

The design for EV-based GAC biomarker discovery is shown in **Figure 1A**. DIA-based quantitative mass spectrometry (12) analysis of 52 samples from the first cohort was conducted and candidate biomarkers was screened through differential protein expression analysis. Logistic regression classification was applied to identify a panel of candidate proteins whose expression were associated with GAC. These biomarkers were further validated by a second cohort of 30 samples.

EV was isolated from the serum using ultracentrifugation (UC). Western blot analysis detected classical EV markers Hsp70 and CD9 in UC fractions, indicating successful enrichment of EV (**Figure 1B**). Particle analysis of randomly selected EV samples showed that the majority of the isolated EV particles ranged between 10~100 nm (**Figure 1C**), which is consistent with the range distribution of exosomes. Furthermore, the morphology of the EV particles as visualized by TEM showed a typical cup-shaped structure with the size between 50~200 nm (**Figure 1D**).

### Proteomic analysis of the EV

**Figure 2:**
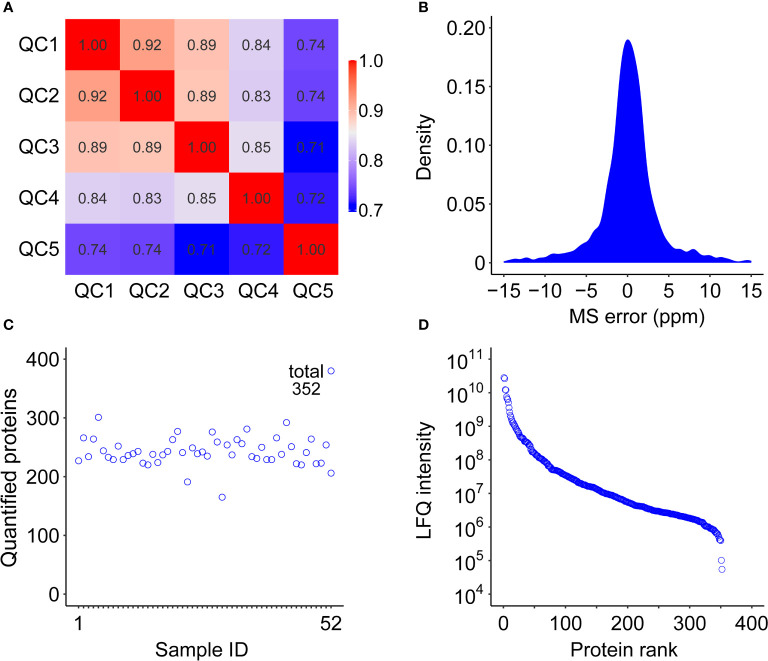
Quality control of the serum EV proteome study from cohort 1. **(A)** Correlation coefficient map of QC samples. **(B)** Distribution of mass error of the identified peptides. **(C)** Numbers of identified proteins in each of the 52 samples in cohort 1. **(D)** Dynamic range of quantified proteins using LFQ (label-free quantification) intensity values.

For quantitative proteomic profiling, EV samples from 19 healthy subjects and 33 GAC patients were analyzed using DIA-based mass spectrometry. To assess the quality of our data as the result of a complex procedure of sample collection and handling, we interspersed quality control (QC) samples during mass spectrometry data acquisition. The distribution of the correlation coefficients of all the QC samples was between 0.71 and 0.92 (**Figure 2A**), indicating reasonable reproducibility. Signal intensity of mass spectrum spanned a dynamic range of six orders of magnitude, with the majority of precursor mass accuracy within ± 5 ppm (**Figures 2B, D**), indicating that our analysis achieved high accuracy and depth.

In total, we identified 448 proteins and quantified 352 proteins from 52 EV samples from cohort 1 (**Figure 2C**). We also performed EV enrichment on cohort 2 of 12 GAC patients and 18 healthy controls, followed by quantitative proteomic analysis, resulting in quantification of 321 proteins. A total of 249 proteins were quantified in both cohorts (**Supplemental Figure 1A**). Gene ontology analysis showed that more than 65% of these proteins localized in extracellular region and exosomes (**Supplemental Figure 1C**), further confirming the successful enrichment of EV.

### Proteomic profiles of serum EV samples from cohort 1

**Figure 3:**
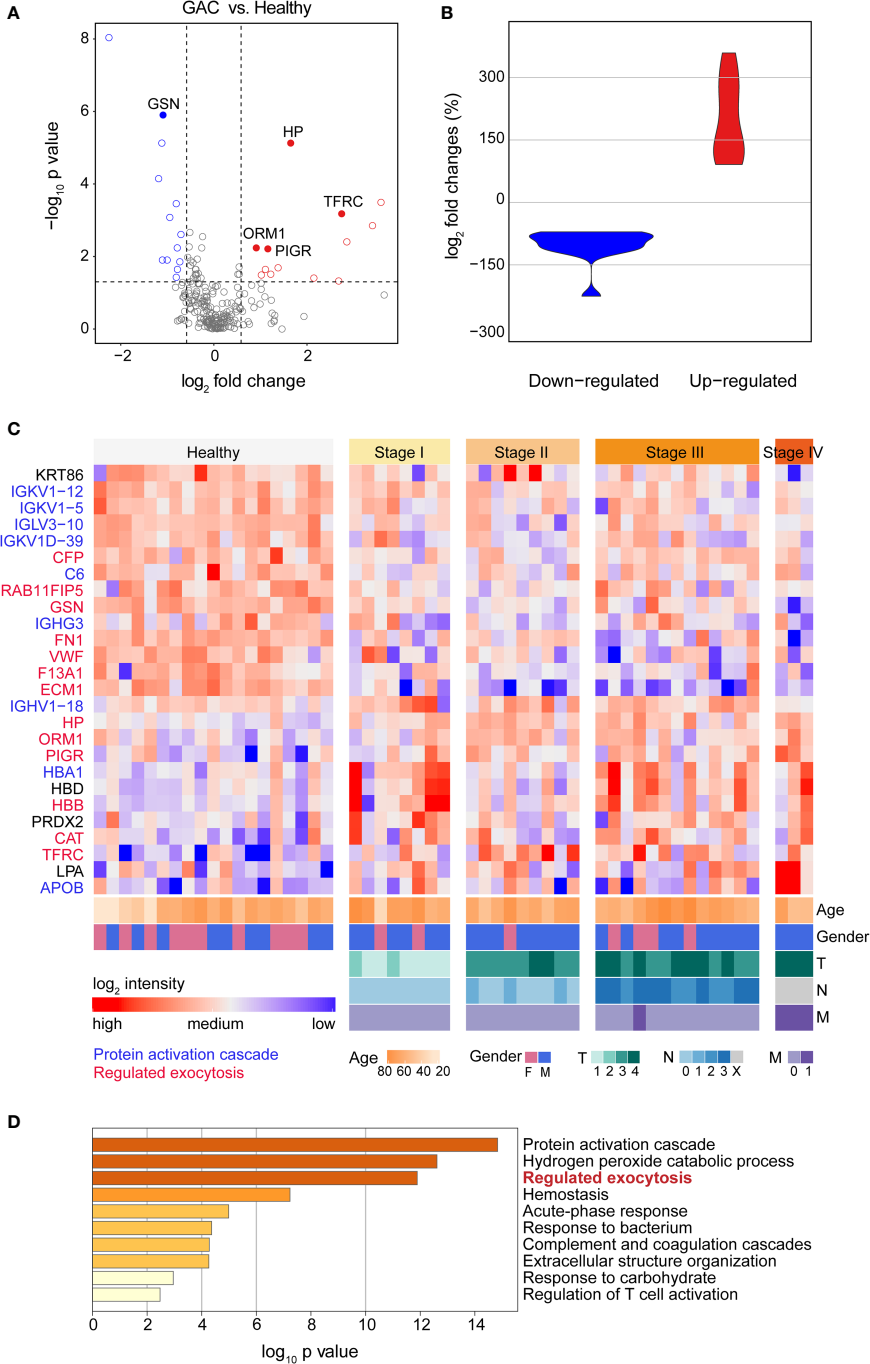
Proteomic profiles of serum EV proteins between heathy subjects and GAC patients. **(A)** Volcano plot of statistical significance value against log_2_-fold change between GAC patients (N=33) and heathy controls (N=19) from cohort 1, showing differentially expressed proteins in blue (down) or red (up) circles. **(B)** Violin plot showing fold changes of up- and down-regulated proteins. **(C)** Heat map of 26 differentially expressed proteins between GAC patients and healthy subjects. Intensities of proteins were log_2_-transformed. Different color in protein names indicates different biological processes derived from these proteins. **(D)** Gene Ontology (GO) analysis of differentially expressed proteins between GAC patients and healthy controls.

Comparing the EV proteome profiles of GAC patients and healthy controls from cohort 1, we found a total of 26 significantly changed proteins, among which 13 were up-regulated and 13 down-regulated (**Figure 3A**). The fold change of up-regulated proteins had a wider range compared to that of down-regulated proteins (**Figure 3B**). The heatmap of differentially expressed proteins displayed distinct patterns, with gender, age and TNM stages of GAC were displayed together (**Figure 3C**). GO analysis showed that the differentially expressed proteins mainly involved in protein activation cascade, hydrogen peroxide catabolic process, regulated exocytosis, hemostasis, acute-phase reaction, response to bacterium (**Figure 3D**). Regulated exocytosis is highly correlated with the secretion of EVs, and contains up-regulated proteins including apolipoprotein B (APOB), haptoglobin (HP), hemoglobin subunit alpha (HBA1) and hemoglobin submit beta (HBB). On the other hand, the majority of the down-regulated proteins involved in complement and coagulation cascade including von Willebrand factor (VWF), coagulation factor XIII A chain (F13A1) and component 6 (C6).

### Discovery of a serum EV biomarker panel for GAC diagnosis

**Figure 4:**
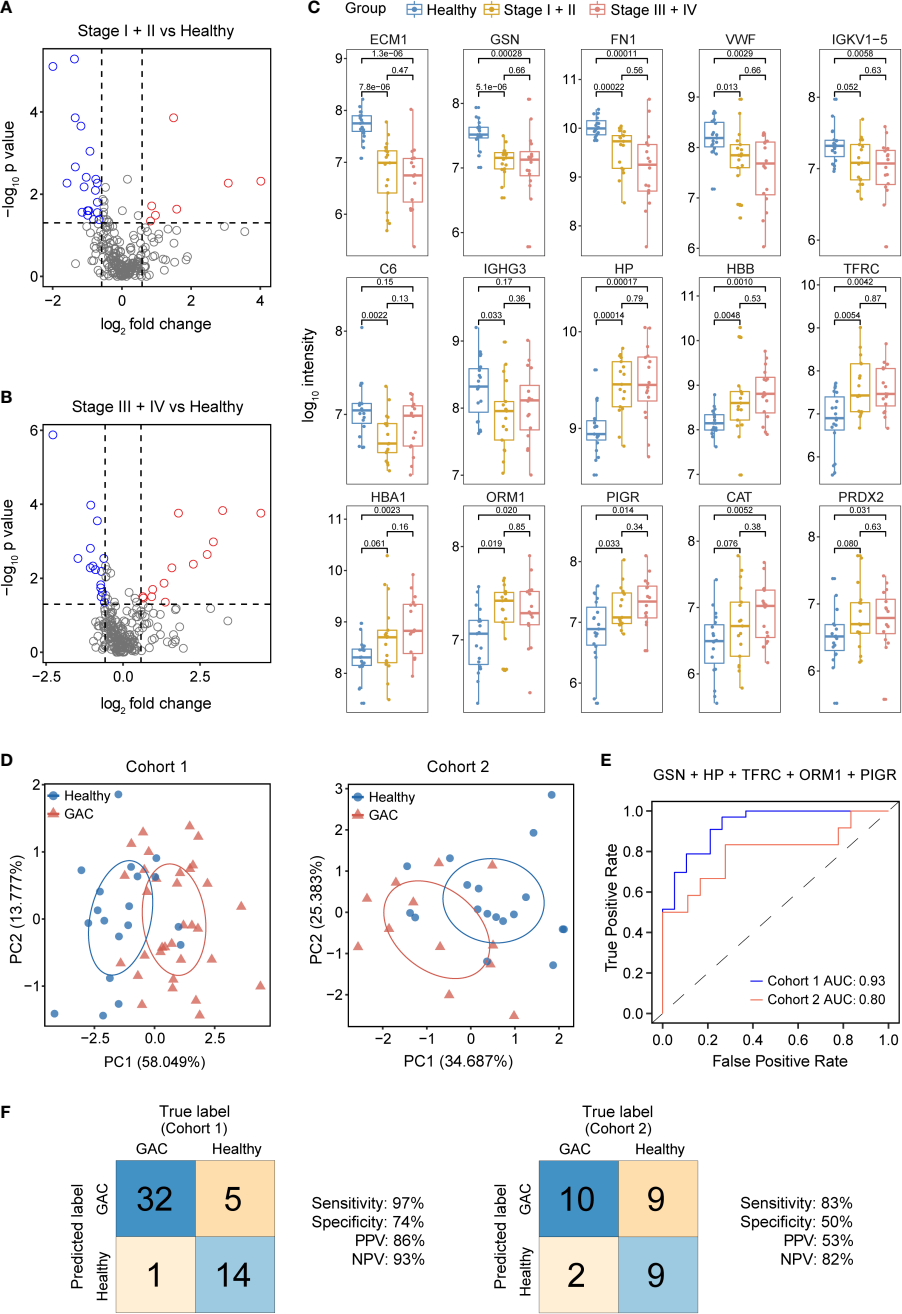
Discovery and validation of serum EV biomarkers for GAC diagnosis. **(A)** Volcano plot of significance value against log_2_-fold change between stage I + II GAC patients (N=17) and heathy controls (N=19) from cohort 1, with significantly changed proteins shown in blue or red circles. **(B)** Volcano plot of significance value against log_2_-fold change between stage III + IV GAC patients (N=16) and heathy controls (N=19) from cohort 1. **(C)**. Box-whisker and dot plots showing distribution of intensity values of 15 candidate proteins across three groups from cohort 1: healthy controls (N=19), GAC stage I + II (N=17) and GAC stage III + IV (N=16). **(D)** Principal component (PC) analysis of healthy control and GAC samples from cohort 1 (left) and cohort 2 (right) using 5 candidate proteins (GSN, HP, TFRC, ORM1 and PIGR). **(E)** ROC curves of the 5-protein logistic regression classifier for GAC diagnosis in cohort 1 and cohort 2. AUC, area under the curve. **(F)** Classification error matrix of the 5-protein logistic regression classifier from E in cohort 1 and cohort 2. The number of samples is noted in each box.

To identify biomarkers with an increased accuracy to differentiate GAC from normal subjects, we performed multi-group differential protein expression analysis using the proteomic data from cohort 1. In addition to comparing GAC patients with healthy controls, patients with early-stage GAC (stage I + II, N=17) and late-stage GAC (stage III + IV, N=16) were also compared to heathy controls (**Figures 4A, B**), and the proteins with high consistency of expression trend were selected as candidate markers. Venn diagram showed the aforementioned three comparisons (**Supplemental Figure 2**). In total, there are 23 intersecting proteins, of which 15 are EV proteins (**Figure 4C**). These 15 proteins were then used as candidate serum EV biomarkers for GAC diagnosis (**Table 2**).

**Table 2:** Expression data of 15 candidate protein markers.

Gene name	Protein name	Fold change			p value		
		GC/HH	S1/HH	S2/HH	GC/HH	S1/HH	S2/HH
ECM1	Extracellular matrix protein 1	0.21	0.25	0.21	9.2E-09	7.8E-06	1.3E-06
GSN	Glycine N-acyltransferase	0.47	0.39	0.56	1.3E-06	5.1E-06	2.8E-04
FN1	Fibronectin	0.46	0.44	0.48	7.5E-06	2.2E-04	1.1E-04
VWF	von Willebrand factor	0.52	0.68	0.36	8.4E-04	1.3E-02	2.9E-03
IGKV1-5	Immunoglobulin kappa variable 1-5	0.58	0.62	0.54	5.8E-03	5.2E-02	5.8E-03
C6	Complement component C6	0.46	0.39	0.54	1.3E-02	2.2E-03	1.5E-01
IGHG3	Immunoglobulin heavy constant gamma 3	0.57	0.50	0.64	3.7E-02	3.3E-02	1.7E-01
HP	Haptoglobin	3.14	2.82	3.48	7.5E-06	1.4E-04	1.7E-04
HBB	Hemoglobin subunit beta	12.05	16.19	7.65	3.2E-04	4.8E-03	1.0E-03
TFRC	Transferrin receptor protein 1	6.71	8.42	4.87	6.6E-04	5.4E-03	4.2E-03
HBA1	Hemoglobin subunit alpha	7.24	7.80	6.66	4.0E-03	6.1E-02	2.3E-03
ORM1	Alpha-1-acid glycoprotein 1	1.88	1.82	1.95	5.8E-03	1.9E-02	2.0E-02
PIGR	Polymeric immunoglobulin receptor	2.23	1.96	2.52	6.2E-03	3.3E-02	1.4E-02
CAT	Catalase	2.60	3.00	3.00	2.0E-02	7.6E-02	5.2E-03
PRDX2	Peroxiredoxin-2	2.03	2.29	1.91	3.2E-02	3.1E-02	3.1E-02

GC, GAC patients; HH, healthy controls; S1, GAC patients in stage I + II; S2, GAC patients in stage III + IV.

For construction of GAC diagnostic models, panels containing 2 to 6 proteins were randomly selected from the 15 candidate proteins through an exhaustive method, and resulting in a total of 9828 combinations. Using cohort 1 as the training set, we built a logistic regression classification model for each panel and calculate the sensitivity, specificity, positive predictive value (PPV), and negative predictive value (NPV). To reduce the false negative predictions, we set the sensitivity greater than 0.9 and retained 2774 classifiers.

We then used Cohort 2 as the testing set to assess the classification accuracy of the models, and found a five-protein panel containing glycine N-acyltransferase (GSN), transferrin receptor protein 1 (TFRC), alpha-1-acid glycoprotein 1 (ORM1), haptoglobin (HP), and polymeric immunoglobulin receptor (PIGR) that showed the best classification performance. This panel of classifiers showed an accuracy, sensitivity, NPV and AUC of 0.97, 0.93 and 0.93 respectively in the training set (**Figures 4E, F**). In the validation set, the sensitivity, NPV and AUC were all above 0.8, indicating that the classifier maintained a good classification performance on new data set. The parameter of the logistic regression model is displayed in **Table 3**. Principal component analysis (**Figure 4D**) also confirmed the effectiveness of the five proteins in distinguishing GAC from healthy samples.

**Table 3:** The five-protein logistic regression classifier for GAC diagnosis.

Protein (Human)	Coefficient	95% CI	p value
HP	3.745	(0.751, 7.545)	0.02601048
PIGR	-0.956	(-3.713, 1.668)	0.47226928
ORM1	-0.206	(-4.143, 3.334)	0.91019726
TFRC	2.070	(0.341, 4.608)	0.05118695
GSN	-3.817	(-8.158, -0.813)	0.03281620
Constant	-12.609	(-56.222, 33.111)	0.56981117

### Discovery of a serum EV biomarker panel for diagnosis of advanced stage GAC

**Figure 5:**
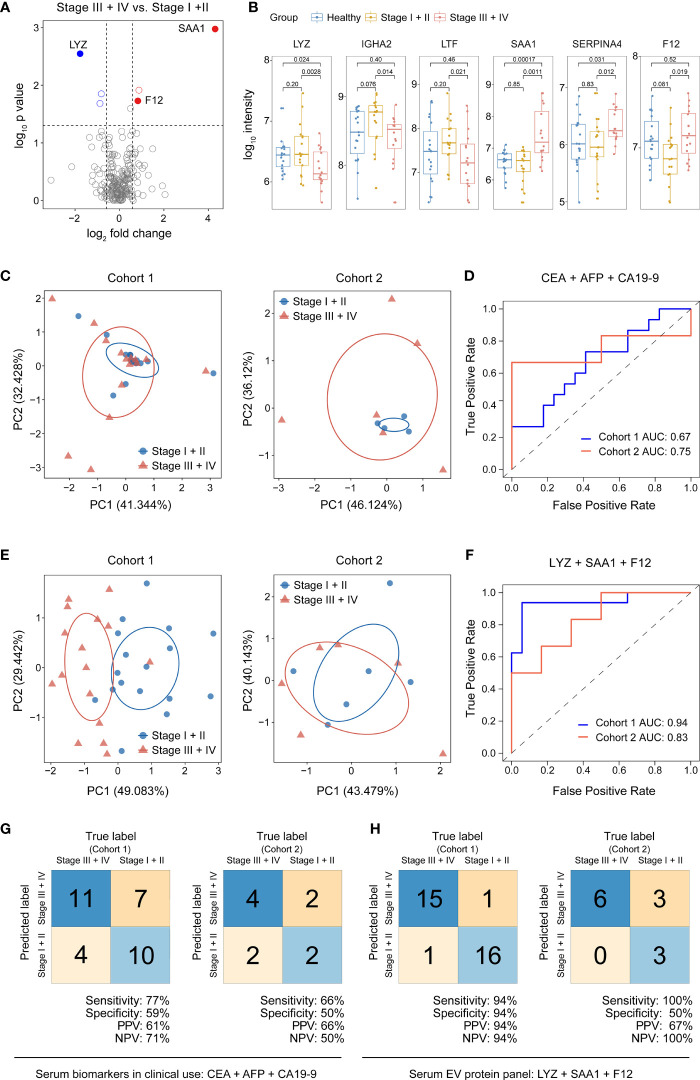
Discovery and validation of serum EV biomarkers for diagnosis of advanced stage GAC. **(A)** Volcano plot of significance values against log_2_-fold change between stage III + IV (N=16) and stage I + II (N=17) GAC patients from cohort 1, showing significantly changed proteins in blue or red circles. **(B)** Box-whisker and dot plots showing distribution of intensity values of 6 candidate proteins across three groups from cohort 1: healthy controls (N=19), stage I + II (N=17) and stage III + IV GAC patients (N=16). **(C)** Principal component (PC) analysis of healthy and GAC samples from cohort 1 (left) and cohort 2 (right) using clinically used serum proteins (CEA, AFP and CA19-9) for diagnosis of advanced GAC. **(D)** ROC curves of the 3-protein (CEA, AFP and CA19-9) logistic regression classifier for diagnosis of advanced GAC. **(E)** Principal component (PC) analysis of healthy controls and GAC samples from cohort 1 (left) and cohort 2 (right) using 3 serum EV proteins (LYZ, SAA1 and F12) for diagnosis of advanced GAC. **(F)** ROC curves of the 3-protein (LYZ, SAA1 and F12) logistic regression classifier for diagnosis of advanced GAC. **(G)** Classification error matrix of the 3-protein logistic regression classifier from D in cohort 1 and cohort 2. **(H)** Classification error matrix of the 3-protein logistic regression classifier from F in cohort 1 and cohort 2. In both **(G, H)**, the number of samples is noted in each box.

Since lymph node is a frequent tumor metastatic site, lymph node metastasis (LNM) is highly informative in selection of treatment strategies (13, 14). At present, the most commonly used blood-based diagnostic markers for GAC in clinic usage are the universal tumor markers carcinoembryonic antigen (CEA), carcinoembryonic antigen (CA19-9, CA72-4, CA24-2, CA50, CA125), and alpha-fetoprotein (AFP) (15–17). Therefore, we used CEA, CA19-9, and AFP as a panel to construct a classifier for diagnosis of advanced GAC, since these proteins are routinely measured in our patients. Based on the patient information in cohort 1, we combined patients in stage I and II as non-LNM group, and patients in stage III + IV as LNM group. In order to distinguish LNM from non-LNM groups, logistic regression was applied using cohort 1 as training set and cohort 2 as validation set, which resulted in a sensitivity, NPV and AUC of 0.66, 0.50 and 0.75 in validation set (**Figures 5C, D, G** and **Table 4**).

**Table 4:** The three-protein logistic regression classifier for diagnosis of advanced stage in GAC.

Protein (Human)	Coefficient	95% CI	p value
CA199	0.008	(-0.001, 0.025)	0.1674706
AFP	0.009	(-0.137, 0.135)	0.8883499
CEA	0.034	(-0.034, 0.12)	0.3503110
Constant	-0.796	(-2.093, 0.396)	0.2022071

In contrast, we used our EV proteomic data to discover potential biomarkers for diagnosis of advanced GAC. We performed differential protein expression analysis comparing patients with stage III + IV (LNM) to that of stage I + II (non-LNM), as shown in **Figure 5A**. We identified 6 differentially expressed proteins including 3 up-regulated proteins and 3 down-regulated proteins (**Figure 5B** and **Table 5**), among which serum amyloid A (SAA1) and immunoglobulin heavy constant alpha 2 (IGHA2) play key roles in receptor-mediated endocytosis. These 6 proteins were used as candidate biomarkers, from which 2 to 5 proteins were randomly selected as panels to construct logistic regression classifiers. Fifty-six classifiers were constructed and trained with cohort 1 to evaluate the classification performance. Then 15 panels were retained with a cutoff of 0.9 for sensitivity and NPV. Applying these classifiers to cohort 2, we found an EV protein panel consisting of lysozyme (LYZ), SAA1, and coagulation factor XII (F12) that showed the best performance, with a sensitivity of 1, NPV of 1 and AUC of 0.83 (**Figures 5E, F, H** and **Table 6**).

**Table 5:** Expression data of 6 candidate protein markers for diagnosis of advanced GAC.

Gene name	Protein name	Fold change	p value
LYZ	Lysozyme	0.29	2.8E-03
IGHA2	Immunoglobulin heavy constant alpha 2	0.57	1.4E-02
LTF	Lactotransferrin	0.54	2.1E-02
SAA1	Serum amyloid A	19.79	1.1E-03
SERPINA4	Serpin family A member 4	1.83	1.2E-02
F12	Coagulation factor XII	1.77	1.9E-02

**Table 6:** The three-protein logistic regression classifier for diagnosis of advanced GAC.

Protein (Human)	Coefficient	95% CI	p value
SAA1	2.577	(0.814, 5.842)	0.03063643
F12	3.719	(0.301, 8.446)	0.06144730
LYZ	-3.818	(-9.187, -0.471)	0.07836439
Constant	-19.814	(-60.292, 12.35)	0.25727669

## Discussion

Based on analysis of protein expression in serum EV and exhaustive feature selection, this study identified a 5-protein panel consisting of GSN, PIGR, TFRC, ORM1, and HP that classifies GAC samples from healthy controls with high accuracy, warranted for further validation. These proteins have been reported in literature and have shown various connections to cancer. GSN is a tumor suppressor down-regulated in gastric cancer cells and gastric tumor tissues, and is a potential therapeutic target (18). TFRC is highly expressed in H. pylori-positive tissues and is a potential indicator for gastrointestinal metaplasia (19). The expression of PIGR is associated with the prognosis of gastric adenocarcinoma, esophageal carcinoma, endometrial carcinoma, hepatocellular carcinoma, and other tumors (20). ORM1 plays an important role in acute phase reaction and inflammatory response, and is highly expressed in plasma of multiple cancers, including gastric cancer (21). HP is the main glycoprotein in the acute phase response, and abnormal glycosylation is associated with several cancers and inflammatory diseases (22). Our study showed that a logistic regression model utilizing these five proteins largely improved the accuracy of distinguishing GAC from healthy subjects.

Immunohistochemistry (IHC) provides valuable information on protein expression profiles in tumor tissues. Although no IHC experiment is conducted in this study, incidentally, we found that in Human Protein Atlas (HPA) database, there are IHC data on all of the marker proteins discovered in our study, in normal and gastric cancer tissues. The data shows that the expression of GSN (https://www.proteinatlas.org/ENSG00000148180-GSN/pathology/stomach+cancer#ihc) is down-regulated in gastric cancer tissues, while TFRC (https://www.proteinatlas.org/ENSG00000072274-TFRC/pathology/stomach+cancer#ihc) and PIGR (https://www.proteinatlas.org/ENSG00000162896-PIGR/pathology/stomach+cancer#ihc) are upregulated in gastric cancer tissues.

The model based on the panel of LYZ, SAA1, and F12 has the potential to identify advanced GAC. Comparing to known protein biomarkers in clinical use, the sensitivity, NPV and AUC of our panel are clearly improved. These three proteins have also been documented in literature. Elevated concentration of SAA1 is associated with occurrence, recurrence and survival of gastric cancer (21). LYZ is associated with incidence of colorectal cancer and lymph node metastasis (23). F12 is a plasma protease which promotes the production of inflammatory bradykinin by activating the kallikrein-kinin system (24). Nevertheless, the specificity and PPV of this model were dramatically decreased in testing data set. We could not rule out the possibility of overfitting due to limited sample size, and further studies with much increased sample size could be the key to address this issue.

In addition to the relatively small sample size, limitation of this study includes the apparent age discrepancy between patients and healthy controls in cohort 1. To rule out the possibility of protein expression changes due to aging, we removed some patients with extremely high ages in cohort 1 to make the median age matching that of the control group and performed differential protein expression analysis. The result shows that the protein markers in our model remains differentially expressed (**STable 1**). In addition, there were essentially no age difference between case and control groups in the validation cohort. Thus, we have strong reason to believe that the age difference between the two groups in cohort 1 was not the major contributing factor for the differential protein expression, which is the basis for our selection of marker proteins. In conclusion, the abnormal expression of these marker proteins appears to have strong association with the growth and progression of GAC tumors, and has the predictive value for identifying GAC at early stage. Further validation of these proteins with increased sample size is warranted.

## Data availability statement

The original contributions presented in the study are included in the article/**Supplementary Material**, further inquiries can be directed to the corresponding authors. The mass spectrometry raw data are available *via* ProteomeXchange with identifier PXD027535.

## Ethics statement

The studies involving human participants were reviewed and approved by the Institutional Reviewing Board of The Ten’s People’s Hospital of Tongji University. The patients/participants provided their written informed consent to participate in this study.

## Author contributions

LL conceived the concept and directed the research. JC performed the experiments and YY analyzed the data. YT, and YZ contributed to some experimental data. LG and DL contributed to clinical samples. JJ contributed to clinical consultation. LL and JC wrote the manuscript. All authors contributed to the article and approved the submitted version.

## Funding

This work was supported by Shanghai Natural Science Foundation grant to LL (19ZR1416400 and 19JC1411900); the East China Normal University National Natural Science Foundation of China grant No. 82273390 to LL (11300-120215-10321).

## Conflict of interest

The authors declare that the research was conducted in the absence of any commercial or financial relationships that could be construed as a potential conflict of interest.

## Publisher’s note

All claims expressed in this article are solely those of the authors and do not necessarily represent those of their affiliated organizations, or those of the publisher, the editors and the reviewers. Any product that may be evaluated in this article, or claim that may be made by its manufacturer, is not guaranteed or endorsed by the publisher.

## Supplementary material

Supplementary Figure 1 Proteome data assessment of the two cohort. **(A)** Venn diagram of identified proteins in training cohort (cohort 1) and testing cohort (cohort 2). **(B)** Venn diagram of the identified proteins with the Vesiclepedia database. **(C)** Gene ontology analysis of shared EV proteins between training cohort and testing cohort.

Supplementary Figure 2 Venn diagrams of differentially expressed proteins obtained in multi-group differential protein expression analysis for cohort 1. **(A)** Venn diagram of down-regulated proteins. **(B)** Venn diagram of up-regulated proteins. GC, GAC patients; HH, healthy individuals; S1, GAC patients of stage I + II; S2, GAC patients of stage III + IV.
