# Supplementary figures and images for: Data-independent acquisition mass spectrometry identification of extracellular vesicle biomarkers for gastric adenocarcinoma
Source: Front Oncol. 2022 Nov 24;12:1051450. doi: 10.3389/fonc.2022.1051450 (PMC9731329; doi:10.3389/fonc.2022.1051450)

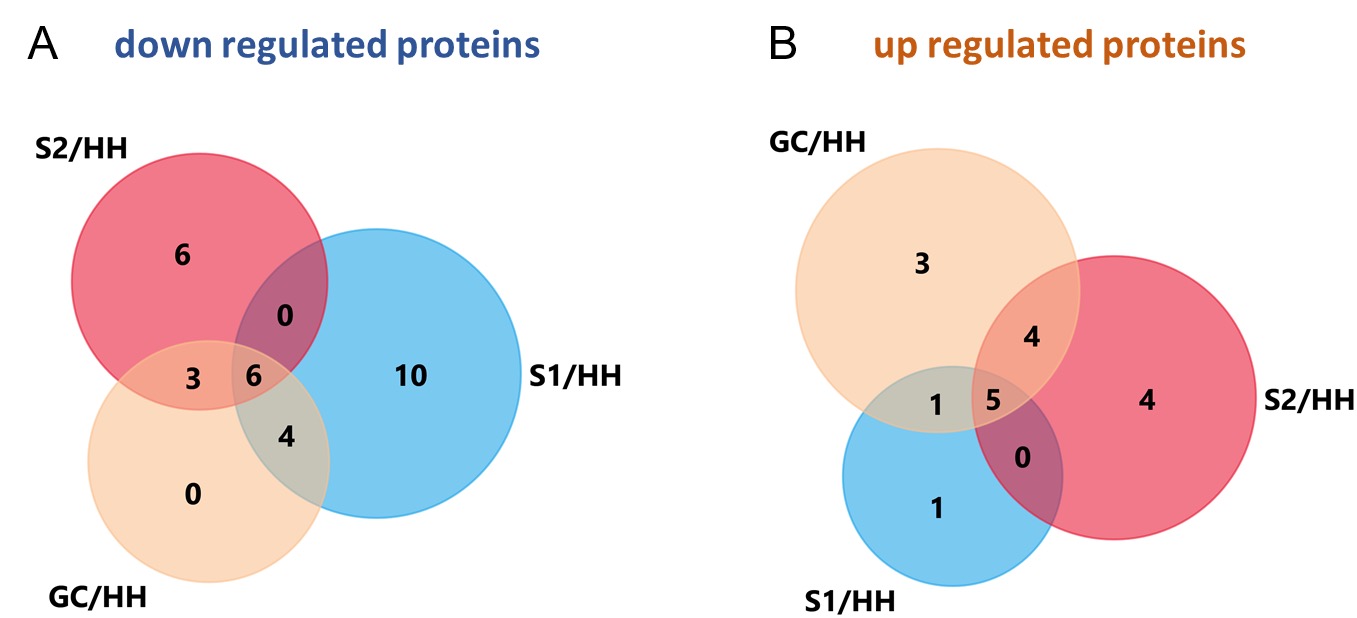

Supplement: Supplementary Figure 1 — Proteome data assessment of the two cohort. (A) Venn diagram of identified proteins in training cohort (cohort 1) and testing cohort (cohort 2). (B) Venn diagram of the identified proteins with the Vesiclepedia database. (C) Gene ontology analysis of shared EV proteins between training cohort and testing cohort. [file DataSheet_1.zip › Fig-S2.jpg]

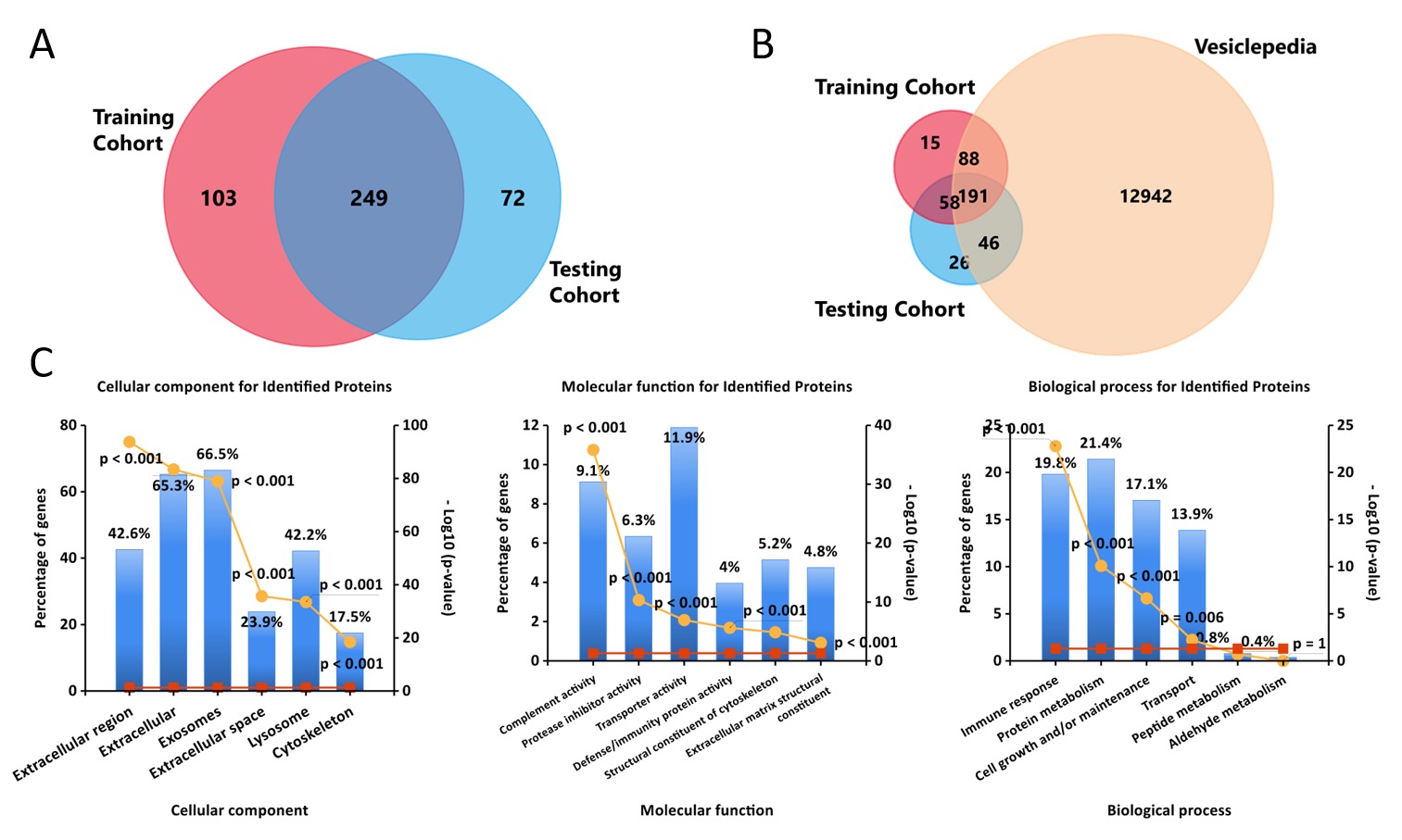

Supplement: Supplementary Figure 1 — Proteome data assessment of the two cohort. (A) Venn diagram of identified proteins in training cohort (cohort 1) and testing cohort (cohort 2). (B) Venn diagram of the identified proteins with the Vesiclepedia database. (C) Gene ontology analysis of shared EV proteins between training cohort and testing cohort. [file DataSheet_1.zip › Fig-S1.jpg]
